# Death

**Published:** 1887-03-26

**Authors:** 


### WORDS OF CONSOLATION.

[Communications for this column should be addressed, "Chaplain," care of the Editor of THE HOSPITAL, who will gratefully welcome any suggestions or inquiries from matrons, nurses, and the staff generally.]

### XXVI.—DEATH.

WE turn our Words of Consolation for awhile into another channel, and invite our readers to some serious meditation upon the subject of death.

Why should we fear death, or why should we not? Hospital suffering and work bring us face to face with it continually. Some who read these lines may know full well how short their time is, and have therefore every reason for desiring to find in their own cases that the "king of terrors" has been robbed of his sting. Others who have longer to wait may solemnly consider whether their views of death are really in accordance with reason and revelation, and therefore reliable. True words on death cannot possibly be a comfort to all. They would not be true if they attempted to be so. The fear of death is common, yet not so common as some people suppose. The ignorant, as a rule, have little fear of it. The poor often welcome it, simply from a desire to be delivered from present misery, not because they have any definite hopes concerning the life to come. Among the better educated some fear death who would not do so were their ideas less morbid, and more in accordance with the Christianity they profess. Others, again, who are very unfit to die, and therefore ought to fear, do not because of their unbelief. More besides these believe, but only tremble when sickness or special circumstances remind them that they are not ready to die.

Let us begin by firmly grasping the Scripture revelation of Death as embodied in the word "sleep." It will be sufficient for the present to refer to the most important passages bearing upon this side of the question. "She is not dead but sleepeth" (Luke viii, 52), was the answer of the Lord of Life to the scorners in Jairus's house. He said this because He knew that the spirit of the child was to be absent from the body but for a little time. Such a brief separation, to be followed by awakening, and by a return to life and health, and the greeting of the family, could not be called death. He said the same of Lazarus, whose spirit was much longer absent. Lazarus had been dead four days; corruption had well-nigh begun, and yet again we have the assurance, "Our friend Lazarus sleepeth, but I go that I may awake him out of sleep" (John xi, 11). Daniel, long before, when shown by the Holy Spirit the certainty of a future resurrection, adopted the same word instead of death: "Many of them that sleep in the dust of the earth shall awake; some to everlasting life, some to shame and everlasting contempt" (Dan. xii, 2). St. Paul carries on the same idea, when speaking of the quick at the second coming of Christ: "We shall not all sleep, but we shall all be changed" (1 Cor. xv, 51). And with regard to the dead in Christ, "Them who sleep in Jesus will God bring with Him" (1 Thess. iv, 14). "That whether we wake or sleep we should live together with Him" (1 Thess. v, 10). The word "death" is purposely avoided in all these passages and "sleep" substituted. We will consider why in our next article.

(*To be continued.*)

